# Laboratory and Genetic Biomarkers Associated with Cerebral Blood Flow Velocity in Hemoglobin SC Disease

**DOI:** 10.1155/2017/6359871

**Published:** 2017-07-16

**Authors:** Rayra Pereira Santiago, Camilo Vieira, Corynne Stephanie Ahouefa Adanho, Sanzio Silva Santana, Caroline Conceição Guarda, Camylla Vilas Boas Figueiredo, Luciana Magalhães Fiuza, Thassila Nogueira Pitanga, Junia Raquel Dutra Ferreira, Milena Magalhães Aleluia, Rodrigo Mota Oliveira, Dalila Luciola Zanette, Isa Menezes Lyra, Marilda Souza Goncalves

**Affiliations:** ^1^Centro de Pesquisas Gonçalo Moniz, Fundação Oswaldo Cruz, Rua Waldemar Falcão, 121, Candeal, 40.296-710 Salvador, BA, Brazil; ^2^Universidade Federal da Bahia, Avenida Adhemar de Barros, s/n, Ondina, 40.170-110 Salvador, BA, Brazil; ^3^Ambulatório Pediátrico de Doença Cerebrovascular, Hospital Universitário Professor Edgard Santos, Universidade Federal da Bahia, Rua Augusto Viana, s/n, Canela, 40110-060 Salvador, BA, Brazil; ^4^Serviço de Pediatria, Hospital Universitário Professor Edgard Santos, Universidade Federal da Bahia, Rua Augusto Viana, sn°, Canela, 40110-060 Salvador, BA, Brazil; ^5^Universidade Salvador, Laureate International Universities, Av. Luís Viana, 3146, Imbuí, 41720-200 Salvador, BA, Brazil

## Abstract

Reference values for cerebral blood flow velocity (CBFV) in hemoglobin SC disease (HbSC) have not been established. We aimed to investigate associations between laboratory and genetic biomarkers associated with CBFV in HbSC children. Sixty-eight HbSC children were included; CBFV was analyzed by transcranial Doppler, and the time-averaged maximum mean velocity (TAMMV) was estimated. Hematological, biochemical, immunological, and genetic analyses were performed. TAMMV was negatively correlated with red blood cell count (RBC) count, hemoglobin, hematocrit, and direct bilirubin (DB), yet positively correlated with monocytes and ferritin. We found that children with TAMMV ≥ 128 cm/s had decreased red blood cell distribution width (RDW) and nitric oxide metabolite (NOx) concentration. Children with TAMMV ≥ 143.50 cm/s had decreased hemoglobin and hematocrit, as well as increased ferritin levels. Decreased hemoglobin, hematocrit, RDW, and NOx and increased ferritin were detected in children with TAMMV ≥ 125.75 cm/s. The CAR haplotype was associated with higher TAMMV. In association analyses, RBC, hemoglobin, hematocrit, RDW, monocyte, DB, NOx, and ferritin, as well as the CAR haplotype, were found to be associated with higher TAMMV in HbSC children. Multivariate analysis suggested that high TAMMV was independently associated with hematocrit, RDW, and NOx. Additional studies are warranted to validate the establishment of a cutoff value of 125.75 cm/s associated with elevated TAMMV in HbSC children.

## 1. Introduction

Sickle cell disease (SCD) is characterized by the presence of hemoglobin S (HbS). The HbSS genotype, in which the beta allele S (*β*^S^) is homozygous, is known as sickle cell anemia (SCA), the most severe type of SCD. In HbS-*β*^0^ thalassemia, another severe form of SCD, the beta allele S is present in association with the absence of synthesis of the *β* gene on the second chromosome. In hemoglobin SC disease (HbSC), there is an association of HbS with another hemoglobin variant, HbC (*β*^C^), that results in a typically milder form of SCD [1].

Clinical complications in HbSC are mild compared to SCA [2, 3]. As hemolysis is less intense in HbSC, anemia is less severe, so complications arising from hemolysis episodes occur less frequently. Nonetheless, individuals with HbSC have an increased risk of acute chest syndrome (ACS), osteonecrosis, and proliferative retinopathy and also face 100 times greater risk of stroke in comparison to the healthy population [2, 4].

The Cooperative Study of Sickle Cell Disease (CSSCD) reported the incidence of stroke at 0.61 per 100 individuals/year in SCA and 0.17 per 100 individuals/year in HbSC [5, 6].

Adams and colleagues [7] established reference cerebral blood flow velocity (CBFV) values using transcranial Doppler (TCD) ultrasonography to identify stroke risk in the context of SCA, but did not attempt to standardize CBFV values related to stroke risk in HbSC [7, 8]. As a result of the lack of studies proposing the establishment of reference values for CBFV in HbSC, physicians have often applied previously determined CBFV reference values for SCA, typically employing the standard proposed by Adams and colleagues [7]. In a previous report that attempted to establish CBFV reference values for individuals with HbSC, Deane and colleagues [9] considered as elevated values those exceeding 128 cm/s. Another study on cerebral velocities in individuals with HbSC reported values lower than those described in individuals with SCA and proposed that velocities above 143.5 cm/s should be considered elevated [10].

Several studies have additionally attempted to investigate associations between elevated CBFV and distinct SCD genotypes, including fetal hemoglobin levels; coinheritance of alpha thalassemia and chronic anemia; leukocyte count; and polymorphisms of methylenetetrahydrofolate reductase (*MTHFR*) 677C>T (rs1801133), Factor V Leiden (*FV*) 1691G>A (rs6025), vascular cell adhesion molecule (*VCAM*) 833T>C (rs1041163), and *VCAM* 1238G>C, although much of this data remains controversial [11–16]. The studies cited above describe the association between abnormal TCD and genetic and laboratory biomarkers exclusively in individuals with SCA, and, to date, the literature contains no published studies attempting to associate specific biomarkers with elevated CBFV values in the context of HbSC. Thus, although HbSC and SCA both fall under the classification of SCD, it is important to note that the clinical course of HbSC disease presents differently than that of SCA.

The absence of well-defined reference values for CBFV and the lack of specific biomarkers in HbSC, combined with the high risk of individuals with SCD to develop neurological clinical manifestations [17, 18], has prompted the search for biomarkers associated with elevated CBFV velocities, especially among children with HbSC, since 54,000 babies with HbSC are born every year, all of whom face severely increased odds of stroke risk [19]. Accordingly, the present study sought to investigate associations between genetic, hematological, immunological, and biochemical laboratory parameters with respect to CBFV in HbSC.

## 2. Materials and Methods

### 2.1. Subjects

The present cross-sectional study included 68 children with HbSC, forty (58.82%) of whom were female, all seen at the Pediatric Cerebrovascular Disease Outpatient Service of the Professor Edgard Santos University Hospital (Federal University of Bahia) from June 2014 to September 2015. The children had an average age of 6.96 ± 3.90 years and a median age of 6.00 years, with a corresponding 25th percentile of 4.00 years and 75th percentile of 9.00 years.

Since all individuals were under 18 years, their legal guardians agreed to biological sample collection procedures and signed terms of informed consent were provided. Children aged 2–17 years with HbSC in a steady state were included. Steady-state HbSC disease is characterized by patients who are not in crisis and those who did not receive blood transfusions in the three months prior to blood collection procedures. Children with a documented previous history of stroke, those undergoing transfusion therapy, and any with hemoglobin profiles divergent from HbSC were excluded.

This study received approval from the Institutional Research Board of the Professor Edgard Santos University Hospital (Federal University of Bahia) (protocol number 287,768/2013) and is in compliance with the Declaration of Helsinki 1964 and its subsequent amendments.

### 2.2. Transcranial Doppler Ultrasonography

All study subjects were submitted to TCD to assess CBFV, which was always performed by a single professional using the same equipment. The time-averaged maximum mean velocity (TAMMV) was measured in the middle cerebral (MCA), anterior cerebral (ACA), and distal intracranial internal carotid (ICA) arteries by a 2 MHz probe through the transtemporal window using a Doppler-Box™ X sonography system (Compumedics Germany GmbH, Singen, Hohentwiel, Germany) [7, 9, 10].

### 2.3. Hematological and Biochemical Parameters

All blood samples were drawn on the same day that TCD was performed, and all blood sampling was performed following no less than 12 hours of fasting.

Hematological parameters were obtained using a CELL-DYN Ruby System hematology analyzer (Abbott Diagnostics, Lake Forest, Illinois, USA), and hemoglobin profiles were analyzed by high-performance liquid chromatography using an HPLC/Variant-II hemoglobin testing system (Bio-Rad, Hercules, California, USA).

Biochemical parameters, including lipid profile, total proteins and fractions, total bilirubin and fractions, lactate dehydrogenase (LDH), alanine transaminase (ALT) and aspartate transaminase (AST), renal profile, and iron, were determined using an automated A25 chemistry analyzer (Biosystems S.A., Barcelona, Catalunya, Spain). Ferritin levels were measured using Access 2 Immunochemistry System (Beckman Coulter Inc., Pasadena, California, USA). C-reactive protein, alpha-1 antitrypsin, and haptoglobin levels were measured using IMMAGE® Immunochemistry System (Beckman Coulter Inc., Pasadena, California, USA).

### 2.4. Nitric Oxide Metabolites

The nitric oxide metabolites (NOx) measurement technique employed herein was performed by a Griess reaction assay and interpreted by spectrophotometry at a wavelength of 560 nm on a SpectraMax 190 Microplate Reader (Molecular Devices Corporation, Sunnyvale, California, USA). All results are expressed in terms of micromolars (*μ*M) of NOx [20].

### 2.5. Genetic Analysis

*Methylenetetrahydrofolate reductase* (*MTHFR*) 677C>T (rs1801133), *Factor V Leiden* (*FV*) 1691G>A (rs6025), *prothrombin* 20210G>A (rs1799963), *vascular cell adhesion molecule* (*VCAM*) 833T>C (rs1041163), and *VCAM* 1238G>C polymorphisms were investigated using polymerase chain reaction (PCR) and restriction fragment length polymorphism (RFLP) techniques [16, 21]. Beta S (*β*^S^) haplotypes were determined by PCR-RFLP [22, 23], and *α*^3.7Kb^ thalassemia (-*α*^3.7^-thal) deletion was assessed by allele-specific PCR [24].

### 2.6. Statistical Analysis

All analyses were performed using the Statistical Package for the Social Sciences (SPSS) v. 20.0 software (IBM, Armonk, New York, USA) and GraphPad Prism version 6.0 (GraphPad Software, San Diego, California, USA). *p* values <0.05 were considered significant. Baseline values of selected variables are expressed as means and stratified according to percentile. The Shapiro-Wilk test was used to determine quantitative variable distribution, and Spearman's rank correlation coefficient measured the strength of linear relationships between paired variables. The Mann–Whitney test and independent *t*-test were used to compare two numerical variables according to distribution. Multivariate binary logistic regression analysis was employed to assess the goodness-of-fit of a model designed to evaluate possible associations between TAMMV and a group of genetic, hematological, and biochemical parameters. The Hosmer-Lemeshow test was used to correct the multivariate analysis. JMP software v.12 (SAS Institute, Cary, North Carolina, USA) was used to assemble correlation graphs.

## 3. Results

The baseline characteristics of the 68 enrolled children with HbSC, including mean ± standard deviation of TAMMV and laboratory parameters, stratified according to the 25th, 50th, and 75th percentiles, are shown in Table 1.

**Figure 1:**
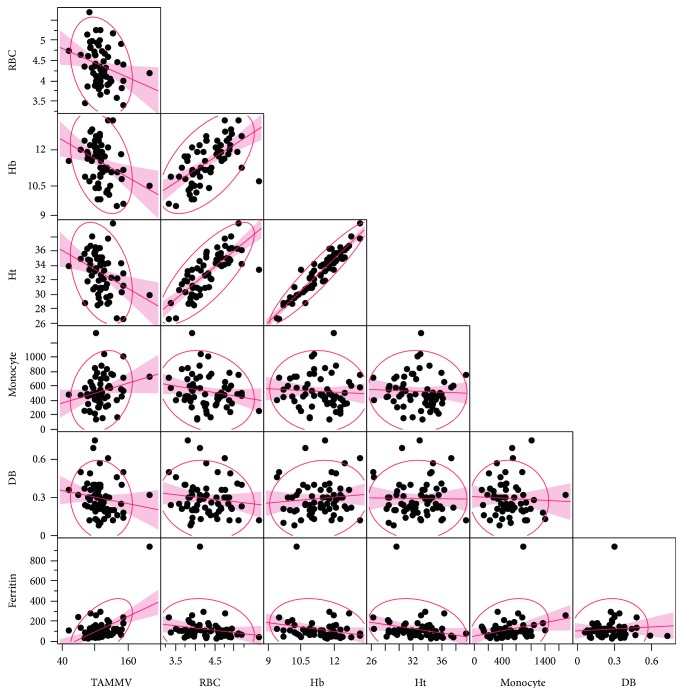
Correlations between TAMMV and systemic biomarkers in children with HbSC. Red blood cells (RBC) (*r* = −0.2734; *p* = 0.0315), hemoglobin (Hb) (*r* = −0.3390; *p* = 0.0070), hematocrit (Ht) (*r* = −0.3470; *p* = 0.0057), and direct bilirubin (DB) (*r* = −0.2545; *p* = 0.0363) are negatively correlated with TAMMV; monocyte count (*r* = 0.2533; *p* = 0.0470) and ferritin (*r* = 0.3044; *p* = 0.0145) are positively correlated with TAMMV.

The median TAMMV was 111.50 cm/s, with a 25th percentile of 101.50 cm/s and a 75th percentile of 125.75 cm/s. TAMMV was negatively correlated with red blood cell (RBC) counts (*r* = −0.2734; *p* = 0.0315), hemoglobin (*r* = −0.3390; *p* = 0.0070), hematocrit (*r* = −0.3470; *p* = 0.0057), and direct bilirubin (DB) (*r* = −0.2545; *p* = 0.0363), yet positively correlated with monocyte counts (*r* = 0.2533; *p* = 0.0470) and ferritin (*r* = 0.3044; *p* = 0.0145) (Figure 1). Figure 1 depicts an outlier who had an abnormal TAMMV of 204.00 cm/s.

Under a protocol designed to establish stroke risk in SCA, previously established by Adams and colleagues [7], two children were considered to have low TCD, 64 had normal TCD, one exhibited abnormal TCD, and one had an inconclusive TCD velocity. It is important to emphasize that this classification is not suitable to assess stroke risk in individuals with HbSC.

**Figure 2:**
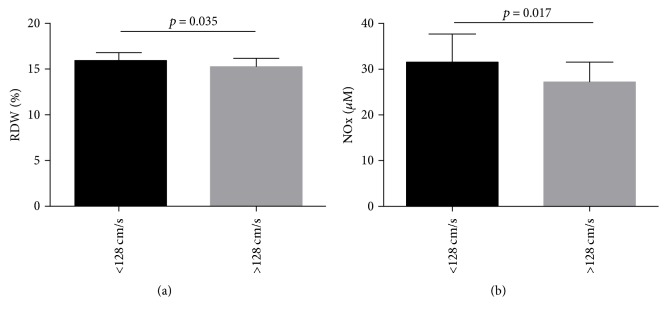
Association of hematological, biochemical, and immunological biomarkers in children with HbSC, using elevated TAMMV as defined by Deane and colleagues (2007). (a) Children with HbSC with TAMMV ≥ 128 cm/s have low RDW (*p* value calculated using Mann–Whitney). The mean and standard deviation were 15.94 ± 1.34% in children with TAMMV < 128 cm/s and 15.26 ± 0.9% in children with TAMMV ≥ 128 cm/s. (b) Children with HbSC with TAMMV ≥ 128 cm/s have low NO metabolite levels (*p* value calculated using *t*-test). The mean and standard deviation were 31.56 ± 6.26 *μ*M in children with TAMMV < 128 cm/s and 27.27 ± 4.09 *μ*M in children with TAMMV ≥ 128 cm/s.

Using a cutoff value of 128 cm/s, previously defined by Deane and colleagues to gauge stroke risk in HbSC [9], 53 children with HbSC were considered to have low TAMMV and 15 children had high TAMMV. A comparison of the hematological, biochemical, and immunological laboratory profiles of these groups found significantly decreased RDW and NOx values in children with HbSC whose TAMMV was higher than 128 cm/s (Figure 2).

**Figure 3:**
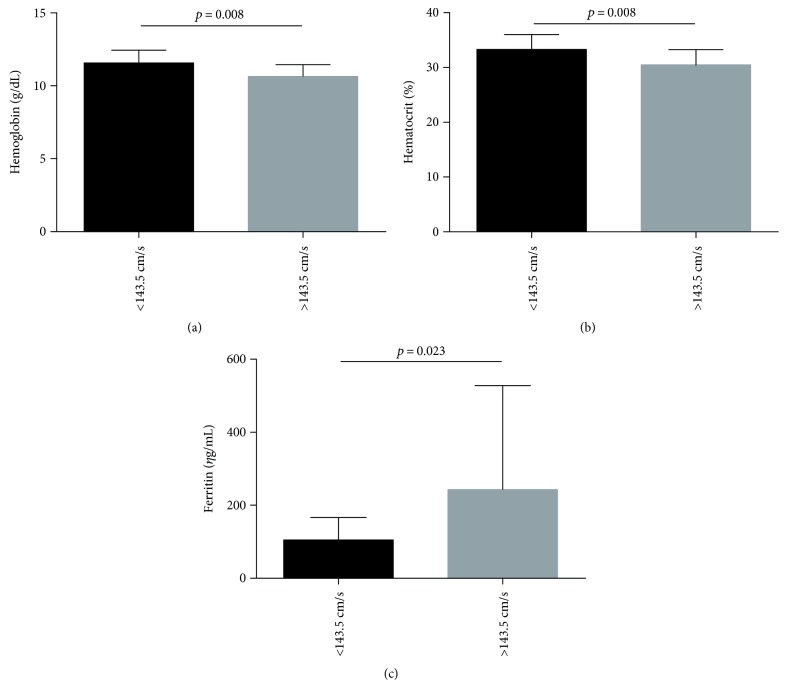
Association of hematological, biochemical, and immunological biomarkers in children with HbSC, using elevated TAMMV as defined by Viera and colleagues (2014). (a) Children with HbSC with TAMMV ≥ 143.50 cm/s have low hemoglobin levels (*p* value calculated using *t*-test). The mean and standard deviation were 11.56 ± 0.89 g/dL in children with TAMMV < 143.5 cm/s and 10.65 ± 0.78 g/dL in children with TAMMV ≥ 143.5 cm/s. (b) Children with HbSC with TAMMV ≥ 143.50 cm/s have low hematocrit levels (*p* value calculated using *t*-test). The mean and standard deviation were 33.32 ± 2.71% in children with TAMMV < 143.5 cm/s and 30.51 ± 2.76% in children with TAMMV ≥ 143.5 cm/s. (c) Children with HbSC with TAMMV ≥ 143.50 cm/s have higher ferritin levels (*p* value calculated using Mann–Whitney). The median and 25th and 75th percentiles were 102.1 (59.53–127.5) *η*g/mL in children with TAMMV < 143.5 cm/s and 151.8 (106.6–224.3) *η*g/mL in children with TAMMV ≥ 143.5 cm/s.

Vieira and colleagues [10] defined a cutoff value of 143.50 cm/s with respect to stroke risk in a sample with HbSC very similar to the present study. Using this value, 60 children with HbSC had low TAMMV, while 8 children had high TAMMV. A comparison of the hematological, biochemical, and immunological laboratory profiles between these groups showed significantly decreased hemoglobin and hematocrit concentrations and increased ferritin levels in children with HbSC whose TAMMV was above 143.50 cm/s (Figure 3).

When a cutoff value corresponding to the 75th percentile of TAMMV was used (125.75 cm/s) to classify children according to stroke risk, 51 were considered to have low TAMMV (i.e., below 125.75 cm/s), while 17 children showed high TAMMV above 125.75 cm/s. Comparing the hematological, biochemical, and immunological profiles of these groups, significantly decreased hemoglobin, hematocrit, RDW, and NOx levels, in addition to elevated ferritin levels, were seen in children with HbSC whose TAMMV was higher than 125.75 cm/s (Table 2).

An analysis of this sample's genetic data revealed the following genotypes: 43 children with a wild-type genotype and 24 heterozygous for the *MTHFR* 677C>T polymorphism; 67 children with a wild-type genotype and one heterozygous for the *FV* 1691G>A polymorphism; 57 children with a wild-type genotype, but 10 were heterozygous for the *VCAM* 833T>C polymorphism, while one presented a variant homozygous genotype for this polymorphism; 61 children with a wild-type genotype and 7 heterozygous for the *VCAM* 1238T>C polymorphism; and 68 children with a wild-type genotype for *PT* 20210G>A. In addition, 53 children were identified with a wild-type genotype of *α*^3.7Kb^ thalassemia, while 13 were heterozygous. Haplotype analyses revealed 35 children with the CAR haplotype and 27 with non-CAR haplotypes (25 BEN haplotype and 2 CAM haplotype). Furthermore, evaluating the haplotypes in the C allele, 50 children were identified with I haplotype, 11 children were identified with II haplotype, and 1 child was identified with III haplotype.

The variant allele of *MTHFR* 677C>T (*p* = 0.524), *VCAM* 833T>C (*p* = 0.546), and *VCAM* 1238T>C (*p* = 0.995) polymorphisms and the presence of *α*^3.7Kb^ thalassemia (*p* = 0.524) as well as the I haplotype of the C allele (*p* = 0.7749) were not found to be associated with high TAMMV. However, the presence of the CAR haplotype was significantly associated with high TAMMV (*p* = 0.038).

Our multivariate analysis model (*p* = 0.004), adjusted for age and sex, was designed to investigate any associations between altered genetic, hematological, and biochemical parameters using the TAMMV value corresponding to the 75th percentile. We found that hematocrit (<33.35%), RDW (≥15.55%), and NOx (<29.86 *μ*M) levels were each independently associated with TAMMV values above the 75th percentile (Table 3).

## 4. Discussion

Although some studies have reported reference values for stroke risk in SCA, the literature contains scarce data concerning similar parameters in HbSC. As relatively few studies have investigated CBFV in individuals with HbSC, we attempted to investigate the existence of associations between genetic, hematological, immunological, and biochemical parameters in children with HbSC presenting elevated TAMMV.

Previous studies have evaluated individuals with HbSC and described an average TAMMV of 94 cm/s and 104.9 ± 19.3 cm/s [9, 10]. In our study, we found that children with HbSC had an average TAMMV of 114.31 ± 22.72 cm/s.

Our correlation analysis found that the children with HbSC who had the highest TAMMV had the lowest RBC, hemoglobin, and hematocrit levels of the sample studied. Accordingly, these children presented more pronounced anemia than those with lower TAMMV. Some authors have reported that anemia may pose an additional risk of stroke development in SCA individuals [7, 13, 25]. It has also been suggested that the increased cerebral blood flow and flow velocity associated with chronic anemia can cause disturbances in blood flow, which may lead to cerebrovascular damage [7, 13, 25]. It is important to highlight that, although anemia tends to be less severe in HbSC than in SCA, the negative correlation described herein suggests that these laboratory parameters related to anemia may be important factors that influence cerebral blood flow [11].

Our correlation analysis also found that the children with HbSC who had the highest TAMMV also had the lowest levels of DB. Decreased levels of DB have been previously described to promote the oxidation of low-density lipoprotein, which leads to generation of reactive oxygen species by cells in addition to being associated with increased atherogenesis risk [26].

In addition, we found that the children with HbSC who had the highest TAMMV had higher monocyte counts and ferritin levels than the remainder of our sample. This finding is supported by a previous study that reported elevated white blood cell counts as a risk factor for a broad range of complications associated with SCD, including stroke, pain crisis, and ACS [27]. In SCD, monocytes exhibit an activated phenotype and are capable of activating endothelial cells [28].

High ferritin levels are commonly described during inflammatory and infectious processes. The observation of high ferritin values present in children with elevated TAMMV could be associated with a chronic inflammatory state and chronic hemolytic condition [29]. However, ferritin levels are highly variable in individuals with SCD [30].

When the TAMMV values from the children with HbSC were analyzed using the cutoff value of 128 cm/s defined by Deane and colleagues [9], lower NOx and RDW levels were found to be associated with TAMMV ≥ 128 cm/s in comparison to those with TAMMV < 128 cm/s. This finding could be explained by intravascular hemolysis, resulting from the release of hemoglobin and arginase into the vascular microenvironment, which rapidly degrade NOx. This cascade of events leads to vasoconstriction and also results in the production of reactive oxygen species in individuals with SCA [31]. This is also consistent with a previous report [32] that demonstrated the importance of continuous NOx production in the maintenance of cerebral blood flow in an experimental model of stroke. Nonetheless, NOx are highly variable in individuals with SCD [33].

RDW, a measurement of RBC size distribution, which could be altered in some types of anemia, is a hematologic parameter that accurately measures the degree of RBC anisocytosis [34]. Although we identified lower RDW values in children with TAMMV ≥ 128 cm/s, these values remain above reference limits, which is consistent with a laboratory classification of anemia [35].

Using the cutoff value of 143.50 cm/s previously proposed by Vieira and colleagues [10], we found an association between lower hemoglobin and hematocrit concentrations, in addition to higher ferritin levels, in children with HbSC whose TAMMV was ≥143.50 cm/s in comparison to those with TAMMV < 143.50 cm/s. This would suggest that children with TAMMV higher than 143.50 cm/s present a more pronounced anemia, a chronic inflammatory state, and a hemolytic condition as discussed above.

Finally, when the cutoff value associated with the 75th percentile (125.75 cm/s) was used to discriminate between low and high TAMMV, we found that lower levels of hemoglobin and hematocrit, lower RDW and NOx concentrations, and higher levels of ferritin were associated with TAMMV ≥ 125.75 cm/s, compared to TAMMV < 125.75 cm/s. Interestingly, these markers were also found to be associated with TAMMVs previously defined by Deane and colleagues [9] and Vieira and colleagues [10].

Thus, children with HbSC with TAMMV lower than those described by Deane and Vieira already present at least some of the hematological and biochemical alterations found when employing the cutoff velocities previously described by these authors [9, 10]. Our results, in turn, seem to suggest that the use of a TAMMV of 125.75 cm/s would be appropriate to identify at-risk individuals with HbSC who undergo routine screening.

Our results show that the variant allele of *MTHFR* 677C>T polymorphism was not associated with high TAMMV. Recent studies have investigated associations between the presence of *MTHFR* 677C>T, *PT* 20210G>A, and *FV* 1691G>A and an increased risk of stroke development in individuals with SCA [11, 36]. Unfortunately, we were unable to detect the variant allele *PT* 20210G>A in any studied individuals and could only identify one individual heterozygous for *FV* 1691G>A, which limits the association between these polymorphisms and TAMMV.

The variant alleles of *VCAM* 833T>C (*p* = 0.546) and *VCAM* 1238T>C (*p* = 0.995) polymorphisms were not associated with high TAMMV. In a previous study, the variant allele of *VCAM* 833T>C polymorphism was not associated with stroke risk when comparing individuals with SCD to healthy individuals; however, the variant allele of the *VCAM* 1238T>C polymorphism has been previously associated with stroke protection [16].

We also found that the *α*^3.7Kb^ thalassemia presence (*p* = 0.524) was not associated with TAMMV. This stands in agreement with previous studies that analyzed *α*^3.7Kb^ thalassemia, which reported no significant associations with TAMMV [27, 37]. Therein, individuals with HbSC were shown to have high blood viscosity, and the presence of *α*^3.7Kb^ thalassemia was associated with increased risk of acute painful episodes, osteonecrosis, and ACS [38].

In addition, we found that the CAR haplotype was associated with high TAMMV. This is consistent with previous studies [11, 39] analyzing the CAR haplotype, which reported an association with increased stroke risk.

The results obtained by our multivariate analysis model suggest the combined influence of the following set of variables when TAMMV was higher than 125.75 cm/s. Hematocrit (<33.35%), RDW (≥15.55%), and NOx (<29.86 *μ*M) levels were each independently associated with TAMMV values above the 75th percentile. This result confirms our findings that individuals with high TAMMV exhibit a more pronounced anemia and hemolytic condition.

Our data highlights the importance of reconsidering appropriate TCD reference values in HbSC. The cross-sectional nature of the present study prevented the establishment of causal inferences, yet it served to confirm the association between laboratory and genetic biomarkers in conjunction with elevated TAMMV in children with HbSC. Further longitudinal studies will be necessary to evaluate the TAMMV cutoff value of 125.75 cm/s (75th percentile) as abnormally high TAMMV and adequate for stroke risk assessment in HbSC. To this aim, we suggest a follow-up with investigation of magnetic resonance imaging, angiography, and neuropsychometric testing. Thereby, we will be able to establish this cutoff value as abnormal by the association with vessel stenosis. In addition, prospective cohort studies also will be needed to more firmly establish associations with clinical outcomes, including ischemic stroke, neurocognitive deficiency, or progression to higher TAMMV and any neurologic impairment that may arise from stroke episodes in these children.

Importantly, a transfusion regimen reduces the HbS levels and increases total Hb [40] and hydroxyurea (HU) therapy [41] increases HbF levels and Hb concentration, which may lead to the prevention of first stroke in children with SCD. These therapeutic approaches should also be investigated in further studies evaluating only children with HbSC.

## 5. Conclusions

The results presented herein suggest that RBC, hemoglobin, hematocrit, RDW, monocyte count, DB, NOx, and ferritin are, in some way, associated with elevated TAMMV in children with HbSC. These markers are known to be involved in inflammation and hemolysis in SCD, and the presence of CAR haplotype has also been associated with elevated TAMMV. Based on our results, we suggest the establishment of a cutoff TAMMV of 125.75 cm/s to assess stroke risk in individuals with HbSC, which nonetheless warrants further investigation that can validate our findings. This TAMMV cutoff value not only is lower than values proposed by previous studies but also has been shown to be associated with altered hematological and biochemical laboratory parameters.

## Figures and Tables

**Table 1:** Baseline characteristics of children with HbSC, including TCD, hematological, biochemical, and immunological data.

Laboratory value	*N*	Mean ± SD	Percentile values		
			25th	50th	75th
TCD					
TAMMV, cm/s	68	114.31 ± 22.72	101.50	111.50	125.75
Hemolysis markers					
RBC, ×10^12^/L	62	4.37 ± 0.49	3.98	4.33	4.75
Hemoglobin, g/dL	62	11.44 ± 0.92	10.80	11.55	12.12
Hematocrit, %	62	32.96 ± 2.86	30.85	33.35	35.02
MCV, fL	62	75.70 ± 5.19	72.12	75.10	79.32
MCH, *ρ*g	62	26.34 ± 2.31	24.87	25.95	27.85
MCHC, g/dL	62	34.76 ± 1.01	34.00	34.90	35.32
RDW, %	62	15.79 ± 1.28	14.70	15.55	16.62
Reticulocyte count, %	61	3.97 ± 1.85	2.75	3.50	4.95
Total bilirubin, mg/dL	68	1.22 ± 1.09	0.60	0.90	1.33
Direct bilirubin, mg/dL	68	0.29 ± 0.13	0.20	0.28	0.36
Indirect bilirubin, mg/dL	68	0.93 ± 1.01	0.38	0.61	0.95
LDH, U/L	65	572.63 ± 185.09	442.00	554.00	663.50
NOx, *μ*M	66	30.63 ± 6.10	25.31	29.86	34.77
Hemoglobin pattern					
Fetal hemoglobin, %	68	2.91 ± 2.28	1.50	2.30	4.02
S hemoglobin, %	68	52.09 ± 2.53	50.40	51.80	53.85
C hemoglobin, %	68	40.59 ± 2.47	39.32	40.70	42.37
A_2_ hemoglobin, %	68	4.33 ± 1.37	3.62	4.20	4.80
Leukocytes					
WBC, ×10^9^/L	62	8249.31 ± 2233.32	6734.50	8080.00	9670.00
Neutrophil count, ×10^9^/L	62	4269.73 ± 1724.12	2753.00	4011.00	5561.50
Segmented count, ×10^9^/L	62	4267.71 ± 1724.47	2753.00	4011.00	5561.50
Eosinophil count, ×10^9^/L	62	434.29 ± 333.50	208.75	349.50	598.00
Basophil count, ×10^9^/L	62	74.84 ± 90.46	0	54.50	106.00
Lymphocyte count, ×10^9^/L	62	2860.61 ± 1068.08	2064.25	2537.50	3522.00
Monocyte count, ×10^9^/L	62	526.18 ± 225.19	383.50	483.00	664.75
Platelets					
Platelet count, ×10^3^/mL	62	251.16 ± 87.93	183.00	230.50	305.75
Platelet volume average, fL	62	7.41 ± 1.84	6.00	7.00	8.52
Glucose					
Glucose, mg/dL	66	75.29 ± 12.21	68.00	75.00	84.25
Lipid metabolism					
Total cholesterol, mg/dL	67	136.24 ± 28.17	121.00	133.00	147.00
HDL-C, mg/dL	65	41.20 ± 9.90	34.00	40.00	48.00
LDL-C, mg/dL	65	80.37 ± 23.52	67.30	78.00	91.20
VLDL-C, mg/dL	68	14.00 ± 4.75	10.20	13.50	17.40
Triglycerides, mg/dL	68	70.00 ± 23.76	51.00	67.50	87.00
Liver					
ALT, U/L	68	16.04 ± 8.67	11.00	14.00	19.00
AST, U/L	68	30.40 ± 10.00	23.00	28.50	36.75
Total protein, g/dL	67	7.28 ± 0.53	6.92	7.26	7.64
Albumin, g/dL	67	4.34 ± 0.32	4.00	4.40	4.60
Globulin, g/dL	67	2.94 ± 0.59	2.50	3.00	3.40
Albumin/globulin ratio	67	1.55 ± 0.41	1.20	1.40	1.90
Iron metabolism					
Serum iron, mcg/dL	65	71.41 ± 23.02	53.45	66.60	88.35
Ferritin, *η*g/mL	64	122.66 ± 120.13	64.00	105.75	134.72
Kidney					
Urea nitrogen, mg/dL	68	19.10 ± 5.57	15.00	19.00	23.00
Creatinine, mg/dL	68	0.52 ± 0.13	0.43	0.50	0.58
Inflammation					
CRP, mg/L	42	3.33 ± 3.11	1.39	2.31	4.35
AAT, mg/dL	48	132.42 ± 32.70	120.50	139.50	150.75
Haptoglobin, mg/dL	48	11.16 ± 17.88	5.83	5.83	5.83

TCD: transcranial Doppler; TAMMV: time-averaged maximum mean velocity; RBC: red blood cells; MCV: mean cell volume; MCH: mean cell hemoglobin; MCHC: mean corpuscular hemoglobin concentration; RDW: red cell distribution width; LDH: lactate dehydrogenase; NOx: nitric oxide metabolites; WBC: white blood cells; HDL-C: high-density lipoprotein cholesterol; LDL-C: low-density lipoprotein cholesterol; VLDL-C: very low-density lipoprotein cholesterol; AST: aspartate aminotransferase; ALT: alanine aminotransferase; CRP: C reactive protein; AAT: alpha 1-antitrypsin; SD: standard deviation.

**Table 2:** Comparison of laboratory data of children with HbSC with TAMMV velocities defined using a cutoff value of 125.75 cm/s (75th percentile).

Laboratory value	TAMMV < 125.75 cm/s		TAMMV ≥ 125.75 cm/s		*p* value^∗^
	*N*	Mean ± SD	*N*	Mean ± SD	
Hemolysis markers					
RBC, ×10^12^/L	46	4.44 ± 0.48	16	4.18 ± 0.49	0.073
Hemoglobin, g/dL	46	11.58 ± 0.81	16	11.04 ± 1.12	**0.042**
Hematocrit, %	46	33.40 ± 2.45	16	31.68 ± 3.59	**0.037**
MCV, fL	46	75.63 ± 5.63	16	75.87 ± 3.77	0.877
MCH, ρg	46	26.30 ± 2.53	16	26.48 ± 1.58	0.783
MCHC, g/dL	46	34.90 ± 0.76	16	34.95 ± 0.79	0.532
RDW, %	46	16.00 ± 1.34	16	15.19 ± 0.90	**0.010**
Reticulocyte count, %	45	4.05 ± 1.94	16	3.74 ± 1.62	0.787^†^
Total bilirubin, mg/dL	51	1.27 ± 1.15	17	1.08 ± 0.89	0.237^†^
Direct bilirubin, mg/dL	51	0.30 ± 0.13	17	0.27 ± 0.14	0.288^†^
Indirect bilirubin, mg/dL	51	0.97 ± 1.08	17	0.80 ± 0.77	0.357^†^
LDH, U/L	50	584.92 ± 201.47	15	531.67 ± 110.50	0.528^†^
NOx, *μ*M	50	31.56 ± 6.36	16	27.75 ± 4.20	**0.029**
Iron metabolism					
Serum iron, mcg/dL	49	69.30 ± 22.89	16	77.88 ± 22.91	0.180^†^
Ferritin, *η*g/mL	47	102.82 ± 62.63	17	177.50 ± 202.97	**0.029** ^†^

RBC: red blood cells; MCV: mean cell volume; MCH: mean cell hemoglobin; MCHC: mean corpuscular hemoglobin concentration; RDW: red cell distribution width; LDH: lactate dehydrogenase; NOx: nitric oxide metabolites; SD: standard deviation; significant *p* values are shown in bold. ^∗^*p* value using *t*-test; ^†^*p* value using Mann–Whitney.

**Table 3:** Multivariable model associating hematologic and biochemical data and gene polymorphisms in TAMMV 75th percentile.

Variables	B	SE	Wald	*p* value	OR	95% CI		*R* square	*p* model
						Lower	Upper		
Model 1									
Hematocrit (<33.35%)	2.95	1.47	4.02	**0.045**	19.25	1.07	346.20	0.432	**0.004**
RDW (≥15.55%)	−3.79	1.50	6.36	**0.012**	0.02	0.00	0.42		
Ferritin (≥105.75 *η*g/mL)	2.09	1.33	2.45	0.117	8.13	0.59	111.87		
NOx (<29.86 *μ*M)	3.33	1.58	4.42	**0.036**	27.99	1.25	625.23		
Absence of *α*^3.7Kb^ thalassemia	−1.53	1.21	1.59	0.206	0.21	0.02	2.32		
CAR haplotype	1.59	1.06	2.25	0.133	4.90	0.61	39.13		
*MTHFR* C677T^∗^	−1.85	1.12	2.71	0.100	0.15	0.01	1.42		
*VCAM* T833C^∗∗^	1.73	1.22	2.01	0.155	5.66	0.51	61.91		
*VCAM* G1238C^∗∗∗^	3.41	1.94	3.10	0.078	30.50	0.68	1368.45		

B: beta coefficient; SE: standard error; OR: odds ratio; CI: confidence interval; RDW: red cell distribution width; NOx: nitric oxide metabolites; ^∗^defined as variant allele T presence; ^∗∗^defined as variant allele C presence; ^∗∗∗^defined as variant allele C presence; TAMMV 75th percentile as a dependent variable defined as TAMMV ≥ 125.75 cm/s; significant *p* values are shown in bold.
